# Incorporation of hazelnut skin green extract in pork burgers delays lipid oxidation during cooking and *in vitro* digestion

**DOI:** 10.1002/jsfa.70039

**Published:** 2025-07-10

**Authors:** Roberta Trovato, Alice Cattivelli, Melissa Zannini, Katia D'Ambra, Giovanna Minelli, Angela Conte, Davide Tagliazucchi, Domenico Pietro Lo Fiego

**Affiliations:** ^1^ Nutritional Biochemistry Lab, Department of Life Sciences University of Modena and Reggio Emilia Reggio Emilia Italy; ^2^ Department of Life Sciences University of Modena and Reggio Emilia Reggio Emilia Italy; ^3^ Interdepartmental Research Centre for Agri‐Food Biological Resources Improvement and Valorisation (BIOGEST‐SITEIA) University of Modena and Reggio Emilia Reggio Emilia Italy

**Keywords:** meat oxidation, phenolic compounds, mass spectrometry, lipid hydroperoxides, flavan‐3‐ols, antioxidants

## Abstract

**BACKGROUND:**

Lipid oxidation occurring during meat cooking and especially digestion has been recently linked with an increased risk of the onset of some chronic diseases. Incorporation of natural antioxidants directly in burger formulations is a novel and captivating approach to enhancing meat quality and repurposing food waste generated by the industry. This research aimed to assess the effect of including a hazelnut skin green extract in pork burgers on lipid oxidation during cooking and during *in vitro* gastro‐intestinal digestion of burgers.

**RESULTS:**

Hazelnut skin green extract was characterized by a high level of phenolic compounds (especially flavan‐3‐ols) that were still present in raw and cooked burgers after hazelnut skin addition. The presence of these compounds reduced the level of lipid hydroperoxides and thiobarbituric acid reactive substances (TBARS) both after cooking and *in vitro* digestion. A significant decrease of 48.6% and 43.3% in lipid hydroperoxides was observed after gastric and intestinal digestion of pork burgers, respectively. Moreover, the inclusion of hazelnut skin green extract decreased by 72.7% the level of TBARS after cooking pork burgers. During *in vitro* digestion, hazelnut skin phenolic compounds were easily released from pork burgers making them potentially available for absorption.

**CONCLUSION:**

The present study demonstrates that the inclusion of by‐products such as hazelnut skin into pork burgers can help to minimize food waste and disposal costs and improve meat quality and human health by delaying lipid oxidation during cooking and digestion. © 2025 The Author(s). *Journal of the Science of Food and Agriculture* published by John Wiley & Sons Ltd on behalf of Society of Chemical Industry.

## INTRODUCTION

Lipid oxidation represents a critical issue, negatively affecting meat quality during storage, cooking, and gastro‐intestinal digestion. The presence of polyunsaturated fatty acids, highly susceptible to oxidation, makes pork and pork‐based products particularly prone to the oxidative phenomena.^1^, ^2^ Lipid degradation leads to the formation of lipid hydroperoxides and advanced lipid oxidation products, such as malondialdehyde and 4‐hydroxy‐2‐nonenal. Once absorbed in the intestine, these compounds can exhibit pro‐atherogenic, cytotoxic and genotoxic effects.^3^, ^4^, ^5^, ^6^ The mechanical handling of meat, including grinding and cooking, leads to the breakdown of cell membranes with consequent exposure of fats to oxygen further intensifying the lipid oxidation process.^7^ In this context, burgers, being a ground product, are particularly vulnerable to oxidative deterioration, resulting in a loss of quality in terms of colour, flavour and texture, as well as the formation of potentially toxic compounds.^8^ These oxidative mechanisms, linked to serious pathologies, including cardiovascular diseases and colorectal cancer, can be strongly accelerated in the gastro‐intestinal environment as a result of the acidic pH of the stomach and the release of free iron and heme during enzymatic digestion.^5^, ^9^ For this reason, several strategies have been exploited to mitigate these effects, including the incorporation of antioxidant compounds in meat and meat products, such as burger, which slow down or inhibit lipid and protein oxidation. Natural antioxidants, derived from plant products, are widely investigated as an alternative to the synthetic ones because they offer a safe and sustainable solution to preserve meat quality. Many natural antioxidants, and in particular phenolic compounds, play a crucial role in preventing lipid oxidation, protecting meat during storage and digestion.^1^, ^2^, ^10^

Hazelnut processing by‐products, such as hazelnut skin, are emerging as a promising solution to prevent lipid oxidation of meat during storage and *in vitro* gastro‐intestinal digestion because of their content in antioxidant compounds, including tocopherols and phenolic compounds.^11^, ^12^ Several studies have investigated the potential applications of hazelnut skin in food industries.^13^, ^14^, ^15^ This by‐product is often treated as a waste, representing an untapped opportunity for their valorization within a circular economy framework. In addition to preventing meat deterioration, phenolic compounds in hazelnut skin may have a positive impact on human health by reducing the risk of oxidative stress‐related diseases, such as cardio‐vascular diseases and colorectal cancer.^16^, ^17^, ^18^

The inclusion of hazelnut skin in pork burgers represents a promising innovation not only for improving the oxidative stability of the product, but also for meeting the demand for healthier and more sustainable food. This approach can help reduce the risk of diseases associated with meat consumption at the same time as enhancing the nutritional quality and sustainability of the products.^1^, ^2^, ^19^

Therefore, the present study aimed to determine the impact of a hazelnut skin green extract (HSGE) inclusion in pork burgers on lipid oxidation during meat cooking and *in vitro* gastro‐intestinal digestion. Furthermore, the phenolic profile of the HSGE, as well as the presence of these compounds in raw and cooked pork burgers and their bioaccessibility during *in vitro* gastro‐intestinal digestion of pork burgers, was assessed by high‐resolution mass spectrometry (HR‐MS).

## MATERIALS AND METHODS

### Materials

All the reagents and enzymes for the *in vitro* gastro‐intestinal digestion and analytical determinations were obtained from Sigma (Milan, Italy). Solvents for extraction and mass spectrometry analysis were purchased from Bio‐Rad (Hercules, CA, USA).

### Preparation and characterization of HSGE

The hazelnut skin was mechanically removed during the roasting process of the hazelnuts. An Italian confectionery company provided this by‐product, whereas the phenolic extract acquired from the same hazelnut skin was prepared by the University of Turin using a subcritical water extraction method as described by Capaldi *et al*.^11^ The nutritional composition (moisture 53 g kg^−1^, lipids 244 g kg^−1^, proteins 60 g kg^−1^, fibers 217 g kg^−1^, saturated fatty acids 92 g kg^−1^, monounsaturated fatty acids 772 g kg^−1^, polyunsaturated fatty acids 136 g kg^−1^) of hazelnut skin was determined following the official methods delineated by the AOAC.^20^

### Pork burger formulation

The pork loin was purchased from a commercial butcher shop in Reggio Emilia (Italy) and was ground in the laboratory as described in a previous paper.^2^ Two burger formulations were created: a control burger consisting of 885 g kg^−1^ longissimus dorsi muscle, 100 g kg^−1^ subcutaneous adipose tissue and 15 g kg^−1^ sodium chloride, as well as another with the addition of 10 g kg^−1^ HSGE. The burgers were moulded using a burger press (50 ± 0.5 g of minced meat, 6 cm in diameter and 1 cm in thickness). The ingredient concentrations were selected based on previous studies by D'Ambra *et al*.^1^, ^2^ demonstrating that the inclusion of HSGE at 1% in pork burgers exerted significant antioxidant effects without altering the organoleptic and sensory properties of the burgers. The initial average pH value of meat was 5.71 ± 0.06. Cooking was performed on a grill plate (Bosch, Gerlingen, Germany) at 180 °C for 3 min (the average internal temperature of the burgers was 88.7 ± 4.5 °C).

### *In vitro* gastro‐intestinal digestion

After cooking, the burgers were cut into small pieces, and *in vitro* digestion was performed on each sample (control and treated) in triplicate, following the INFOGEST 2.0 protocol described in Brodkorb *et al*.^21^ Briefly, 1 g of cooked meat was added to 1 mL of salivary fluid containing salivary *α*‐amylase, achieving a final concentration of 150 U mL^−1^. This mixture was incubated for 2 min at 37 °C in a rotating wheel set at 10 rpm. After this initial incubation, 2 mL of gastric fluid was added, and the pH was adjusted to 3 using 6 mol L^−1^ hydrochloric acid. Pepsin was then incorporated at a final concentration of 2000 U mL^−1^. The mixture was then incubated for 120 min at 37 °C with rotation at 10 rpm. Following the gastric phase, 4 mL of intestinal fluid was added to the bolus. The pH was then raised to 7.5 using concentrated sodium hydroxide, and the chyme was incubated for an additional 30 min at 37 °C, again with rotation at 10 rpm. Finally, intestinal digestion was initiated by adding pancreatin, achieving a final concentration based on trypsin activity of 200 U mL^−1^. The chyme was further incubated for 120 min at 37 °C in the rotating wheel at 10 rpm. At the end of each digestion step, aliquots were immediately stored at −20 °C for subsequent analyses, as described below. To account for possible interferences in the assays, a control digestion with water instead of burger was performed.

### Lipid hydroperoxide extraction and determination

Lipid hydroperoxides were isolated from raw, cooked, and *in vitro* digested burger samples. One gram of finely chopped raw or cooked burger was homogenized with 7 mL of HPLC‐grade methanol containing 4 mmol L^−1^ butylated hydroxytoluene (BHT) using an Ultra‐Turrax (T25‐digital; IKA, Staufen, Germany) at 5000 × *g* for 30 s at 4 °C, repeated three times. Similarly, 50 μL of digested burger homogenates were combined with 450 μL of methanol containing 4 mmol L^−1^ BHT. The mixtures were then subjected to mild agitation for 60 min at room temperature, following the procedure described by Tagliazucchi *et al*.^22^ Afterward, the samples were centrifuged at 3000 × *g* for 15 min at 4 °C, and the supernatants were collected for subsequent determination of lipid hydroperoxides. The quantification of lipid hydroperoxides was performed using the FOX assay at 560 nm adapted to be read using a microplate reader.^23^, ^24^ For the assay, 60 μL of the extracted samples were mixed with 140 μL of FOX reagent [comprising 50 μL of (NH_4_)_2_Fe(SO_4_)_2_ 1 mmol L^−1^ in H_2_SO_4_ 0.1 mol L^−1^, 20 μL of xylenol orange 1 mmol L^−1^, 20 μL of BHT 40 mmol L^−1^, 20 μL of H_2_O and 30 μL of MeOH HPLC‐grade] and incubated at room temperature for 30 min. The lipid hydroperoxides content was expressed as μmol H_2_O_2_ equivalents per kg of meat.

### Determination and quantification of advanced lipoxidation end‐products

Advanced lipoxidation end‐products were measured as thiobarbituric acid reactive substances (TBARS) in raw, cooked, and digested burger samples, following the protocol described by Buege *et al*.,^25^ with slight modifications. For the analysis, 1.25 g of minced raw or 0.625 g of the minced cooked burger were blended with 3.75 mL or 4.375 mL of distilled water, respectively, and homogenized using an Ultra‐Turrax homogenizer (T25‐digital; IKA) for 30 s, repeated three times with 1 min intervals at a speed of 5000 × *g*. Digested samples were directly used without further preparation.

For the assay, 300 μL of the sample were combined with 300 μL of water, followed by the addition of 360 μL of trichloroacetic acid 500 g kg^−1^and 600 μL of thiobarbituric acid (TBA) solution (7.5 g kg^−1^ in 0.5 mol L^−1^ HCl). The resulting mixture was incubated at 80 °C for 60 min in a thermostatic water bath, cooled to room temperature and centrifuged at 10000 × *g* for 10 min at 4 °C. The concentration of TBA‐reactive substances (TBARS) in the supernatant was quantified spectrophotometrically at 532 nm, with results expressed as μmol of malondialdehyde equivalents per kg of meat.

### Extraction of total phenolic compounds from HSGE and pork burgers, and UHPLC/HR‐MS identification

The extraction of phenolic compounds from HSGE was carried out as reported in D'Ambra *et al*.^1^ The extraction of phenolic compounds in raw and cooked burgers was performed by grinding 1.5 g of sample with 2.5 mL of a methanol/water/formic acid solution (70/28/2, v/v). After incubation at 37 °C for 60 min at a constant speed, samples were centrifuged at 3000 × *g* for 20 min at 4 °C, and the resulting supernatant was collected and filtered using a 0.22‐μm syringe filter. The digested samples were directly centrifuged, followed by collection and filtration of the supernatant. Each sample was extracted in triplicate and stored at −20 °C for HR‐MS analysis.

Phenolic compounds were initially separated by UHPLC (Ultimate 3000 separation module) through a C18 column (Acquity UPLC HSS C18 Reversed phase; 2.1 × 100 mm, 1.8 μm particle size; Waters, Milan, Italy) before injection into the HR‐MS system consisting of a Q Exactive Hybrid Quadrupole‐Orbitrap Mass Spectrometer (Thermo Fisher Scientific, Waltham, MA, USA). The chromatographic and mass spectrometry conditions and the full protocol were described in Martini *et al*.^23^ Quantification was carried out by building external calibration curves with the available standard compounds as reported in the Supporting information (Table S1). The bioaccessibility index (BI) was calculated as described in Cattivelli *et al*.^26^

### Statistical analysis

*In vitro* digestion and analytical determinations were carried out in triplicate and the data are presented as the mean ± SD. ANOVA (Univariate analysis of variance) followed by a Tukey's post‐hoc test was carried out via Prism, version 6.0 (GraphPad Software Inc., San Diego, CA, USA). *P* < 0.05 was considered statistically significant.

## RESULTS AND DISCUSSION

### Phenolic compound profile of HSGE

The phenolic profile of HSGE was thoroughly investigated by HR‐MS. The total phenolic content was 40 141.3 ± 1273.5 μmol kg^−1^ HSGE. The phenolic profile of HSGE was dominated by flavan‐3‐ols, which represented 79.2% of total phenolic compounds (Table 1). From a quantitative point‐of‐view, the second most important class of phenolic compounds was hydroxybenzoic acids followed by flavonols, which accounted for 8.5% and 5.1% of total phenolic compounds. Looking at the individual compounds, the flavan‐3‐ols present in the highest amounts were epicatechin (15 463.2 ± 413.9 μmol kg^−1^ extract) and catechin (8383.5 ± 268.5 μmol kg^−1^ extract) followed by three isomers of the oligomeric procyanidin dimer B‐type, one isomer of procyanidin trimer B‐type and epigallocatechin‐3‐*O*‐gallate (Table 1). Appreciable amounts of ellagic acid and quercetin were also detected in HSGE. Furthermore, the most representative hydroxybenzoic acids were gallic acid, one isomer of hydroxybenzoic acid and protocatechuic acid (Table 1). Concerning flavonols, the compounds present in the highest amount were the aglycones quercetin and myricetin, and quercetin‐3‐*O*‐rhmanoside (Table 1).

**Table 1:** Amount of phenolic compounds identified in the hazelnut skin green extract (HSGE) and in raw and cooked pork burgers formulated with HSGE

Compound	HSGE	Raw pork burger	Cooked pork burger
	μmol kg^−1^ HSGE	μmol kg^−1^ meat	μmol kg^−1^ meat
Hydroxybenzoic acid	925.0 ± 32.6	8.2 ± 0.1 a	8.5 ± 0.1 a
Dihydroxybenzoic acid	223.0 ± 15.8	1.7 ± 0.1 a	1.6 ± 0.0 a
Protocatechuic acid	489.7 ± 18.5	4.5 ± 0.2 a	4.7 ± 0.1 a
Gallic acid	858.2 ± 25.4	9.0 ± 0.4 a	9.1 ± 0.0 a
Galloyl‐shikimic acid isomer 1	248.8 ± 6.7	2.3 ± 0.1 a	2.2 ± 0.0 a
Galloyl‐shikimic acid isomer 2	166.5 ± 4.5	1.4 ± 0.1 a	1.4 ± 0.0 a
Gallic acid‐hexoside isomer 1	17.5 ± 0.4	0.6 ± 0.1 a	0.6 ± 0.1 a
Gallic acid‐hexoside isomer 2	73.2 ± 0.4	0.5 ± 0.0 a	0.3 ± 0.2 a
Gallic acid‐hexoside isomer 3	11.4 ± 0.5	0.2 ± 0.0 a	0.2 ± 0.0 a
Gallic acid‐hexoside isomer 4	10.0 ± 0.2	0.1 ± 0.0 a	0.1 ± 0.0 a
Syringic acid‐4‐*O*‐hexoside isomer 1	75.4 ± 1.1	ND	ND
Syringic acid‐4‐*O*‐hexoside isomer 2	48.4 ± 1.3	ND	ND
Syringic acid‐4‐*O*‐hexoside isomer 3	258.9 ± 12.1	2.6 ± 0.1 a	2.9 ± 0.1 a
*Total hydroxybenzoic acids*	*3405.9 ± 119.4*	*30.9 ± 1.1 a*	*31.7 ± 0.8 a*
Epicatechin	15 463.2 ± 413.9	128.4 ± 2.8 a	135.7 ± 1.9 b
Catechin	8383.5 ± 268.5	71.0 ± 2.6 a	78.3 ± 0.8 b
Epigallocatechin	658.7 ± 14.1	5.7 ± 0.3 a	6.1 ± 0.0 a
Gallocatechin	343.9 ± 12.1	3.2 ± 0.1 a	3.4 ± 0.1 a
Epicatechin‐3‐*O*‐gallate	940.0 ± 33.9	ND	8.7 ± 0.1
Catechin‐*O*‐hexoside	8.3 ± 1.0	ND	ND
Epigallocatechin‐3‐*O*‐gallate	24.6 ± 0.6	0.4 ± 0.0 a	0.4 ± 0.1 a
Gallocatechin‐3‐*O*‐gallate	33.6 ± 0.7	ND	ND
Procyanidin dimer A type isomer 1	35.5 ± 4.1	ND	ND
Procyanidin dimer A type isomer 2	57.1 ± 0.9	ND	ND
Procyanidin dimer A type isomer 3	25.2 ± 5.6	ND	ND
Procyanidin dimer B type isomer 1	1024.5 ± 32.7	17.0 ± 0.6 a	18.1 ± 0.4 a
Procyanidin dimer B type isomer 2	1159.2 ± 28.0	15.0 ± 0.5 a	16.5 ± 0.0 b
Procyanidin dimer B type isomer 3	1761.1 ± 18.7	2.9 ± 0.0 a	2.4 ± 0.0 a
Catechin‐gallocatechin isomer 1	329.5 ± 12.5	ND	ND
Catechin‐gallocatechin isomer 2	199.8 ± 6.8	ND	ND
Procyanidin dimer B type gallate isomer 1	99.5 ± 8.2	ND	ND
Procyanidin dimer B type gallate isomer 2	74.1 ± 3.8	ND	ND
Procyanidin trimer B type	1004.3 ± 21.9	ND	ND
Prodelphinidin trimer B type	186.1 ± 4.1	ND	ND
*Total flavan‐3‐ols*	*31 811.4 ± 892.2*	*243.5 ± 6.9 a*	*269.8 ± 3.4 b*
Quercetin	1298.4 ± 45.7	8.3 ± 0.3 a	9.4 ± 0.1 b
Myricetin	244.0 ± 3.9	1.4 ± 0.0 a	1.6 ± 0.0 a
Kaempferol‐3‐*O* rhamnoside	23.9 ± 0.6	0.2 ± 0.0 a	0.2 ± 0.0 a
Quercetin‐3‐*O*‐rhamnoside	372.3 ± 9.2	3.1 ± 0.1 a	3.5 ± 0.0 a
Myricetin‐3‐*O*‐rhamnoside	9.84 ± 0.11	1.0 ± 0.0 a	1.1 ± 0.0 a
Quercetin‐3‐*O*‐rutinoside	10.4 ± 0.6	0.1 ± 0.0 a	0.1 ± 0.0 a
Isorhamnetin‐3‐*O*‐rutinoside	9.2 ± 0.4	0.1 ± 0.0 a	0.1 ± 0.0 a
*Total flavonols*	*2056.6 ± 61.5*	*14.1 ± 0.5 a*	*16.1 ± 0.1 b*
Phloretin 2’‐*O*‐glucoside	384.1 ± 9.4	3.3 ± 0.0 a	3.7 ± 0.1 a
Ellagic acid	2483.4 ± 191.0	6.0 ± 0.3 a	6.4 ± 0.3 b
*Total others*	*2867.4 ± 200.4*	*9.3 ± 0.3 a*	*10.2 ± 0.3 a*
*Total phenolic content*	*40 141.3 ± 1273.5*	*297.8 ± 8.7 a*	*327.7 ± 4.7 b*

*Note*: ND, compound was not detected in the sample. Results are expressed in μmol kg^−1^ HSGE or meat. Different lowercase letters in the same row indicate that the values are significantly different (*P <* 0.05).

A similar phenolic compound profile has already been reported in previous studies.^11^, ^27^, ^28^, ^29^ For example, Del Rio *et al*.^27^ found that flavan‐3‐ols represented about 95% of total phenolic compounds in hazelnut skin, with obvious differences among the samples from different origins. In general, monomeric and oligomeric flavan‐3‐ols dominated the phenolic profiles of hazelnut skin.^11^, ^27^, ^28^, ^29^ In addition, hydroxybenzoic acids such as gallic acid and protocatechuic acid have already been identified as the major phenolic acids in hazelnut skin.^15^, ^28^, ^30^ Moreover, quercetin and quercetin‐3‐*O*‐rhamnoside were reported to be the most important flavonols identified in hazelnut skin.^27^ Nonetheless, some phenolic compounds (such as ellagic acid) detected in appreciable amounts in HSGE were described here for the first time.

Although the phenolic profile of the HSGE analysed in this study was similar to those previously published, some significant qualitative differences are present. It should be noted that in the mentioned previous studies, phenolic compounds were analysed in hazelnut skin after extraction with solvents such as methanol, whereas the present extract was obtained by a subcritical water extraction process, as reported in Capaldi *et al*.^11^

### Effect of the inclusion of HSGE in pork burgers on lipid peroxidation during cooking and *in vitro* digestion

**Figure 1:**
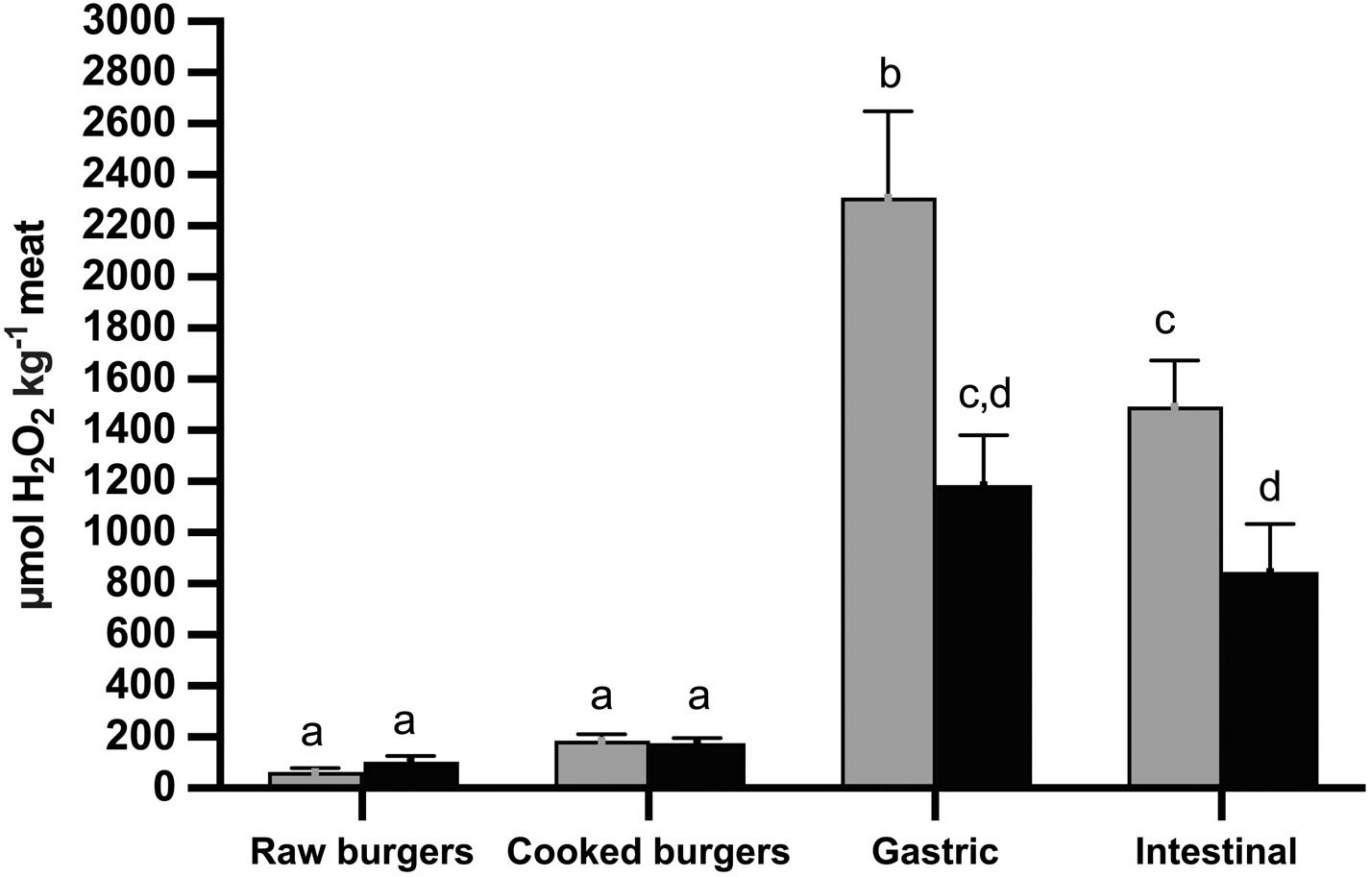
Effect of the inclusion of hazelnut skin green extract on the amount of lipid hydroperoxides in pork burgers before and after gastric and intestinal digestion. Analysis was carried out on raw meat, cooked meat and meat subjected to *in vitro* gastric and intestinal digestion. Results are expressed as μmol of H_2_O_2_ equivalent kg^−1^ meat. Different colours identified the different treatments. Grey bars: control pork burgers; black bars: hazelnut skin green extract supplemented burgers. Different lowercase letters indicate that the values are significantly different (*P* < 0.05).

No significant differences (*P* > 0.05) were found in the concentration of lipid hydroperoxides between the raw pork burger control group (CB) and the burger group added with HSGE (HSGEB) (Fig. 1). The amount of lipid hydroperoxides experienced a threefold increase after cooking in both the CB and HSGEB groups. Once again, no significant differences (*P* > 0.05) were found between the two burger groups (Fig. 1).

*In vitro* gastro‐intestinal digestion of cooked pork burgers from the CB group triggered a substantial increase in the amount of lipid hydroperoxides (Fig. 1). The concentration of lipid hydroperoxides in burgers from the CB group increased from 185.8 ± 26.2 μmol H_2_O_2_ kg^−1^ meat in the cooked burger to 2311.7 ± 337.3 μmol H_2_O_2_ kg^−1^ meat after *in vitro* gastric digestion (Fig. 1). By contrast, at the end of the intestinal phase of the digestion, a decrease of 1.55 times in the amount of lipid hydroperoxides was observed, reaching the final value of 1494.3 ± 179.1 μmol H_2_O_2_ kg^−1^ meat (Fig. 1). Similarly, *in vitro* gastro‐intestinal digestion of high‐fat beef and of turkey meat resulted in an increase in lipid hydroperoxides during gastric digestion followed by a decrease during the intestinal phase.^31^, ^32^

The inclusion of HSGE in burger formulation significantly (*P* < 0.05) decreased the level of lipid hydroperoxides both after gastric and intestinal digestion (Fig. 1). A 48.6% decrease in lipid hydroperoxides was observed after gastric digestion in HSGE‐formulated burgers compared to control burgers. Similarly, the amount of lipid hydroperoxides was 43.3% lower in HSGE‐treated burgers with respect to the CB group after intestinal digestion (Fig. 1).

**Figure 2:**
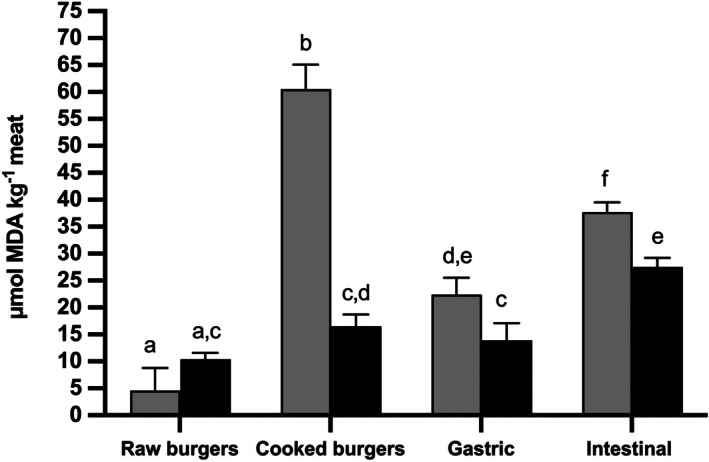
Effect of the inclusion of hazelnut skin green extract on the amount of lipid oxidation end‐products in pork burgers before and after gastric and intestinal digestion. Analysis was carried out on raw meat, cooked meat and meat subjected to *in vitro* gastric and intestinal digestion. Results are expressed as μmol of malondialdheyde (MDA) equivalent kg^−1^ meat. Different colours identified the different treatments. Grey bars: control pork burgers; black bars: hazelnut skin green extract supplemented burgers. Lipid oxidation end‐products were quantified as thiobarbituric acid‐reactive substances (TBARS). Different lowercase letters indicate that the values are significantly different (*P* < 0.05).

The trend of the TBARS values was quite different from that of lipid hydroperoxides. No significant differences (*P* > 0.05) were found between the two burger groups in raw meat (Fig. 2). However, after cooking the amount of TBARS significantly increased (*P* < 0.05) by about 13 times in control burgers. Including HSGE in pork burgers mitigated the increase in TBARS after cooking, resulting in a value lower by about 73% compared to the CB group. A non‐significant 1.6 times increase (*P* > 0.05) in TBARS values was also observed in HSGE‐formulated burgers (Fig. 2). The TBARS values significantly decreased (*P* < 0.05) by 2.7 times after *in vitro* gastric digestion of control burgers and then further increased by about 1.7 times at the end of the intestinal phase of the digestion (Fig. 2). The observed increase in the TBARS values, passing from the gastric to the intestinal digestion, was concomitant with the recorded decrease in lipid hydroperoxides concentration (Figs 1 and 2). Including HSGE in pork burgers resulted in a 38% and 27% decrease in the TBARS values compared to the CB after *in vitro* gastric and intestinal digestion, respectively (Fig. 2).

The inclusion of antioxidant‐rich extracts from foods or by‐products in meat formulation is a recently explored and emerging approach to control and reduce oxidative phenomena affecting lipids during storage, cooking, and digestion. Recently, Ngongoni *et al*.^33^ showed that the addition of *Acacia mearnsii* bark and leaves to ground beef patties reduced the TBARS values during meat storage. Several research papers demonstrated the protective effect of pomegranate peel extract rich in phenolic compounds on lipid oxidation during storage and cooking after inclusion in different types of meat.^34^

Very few studies investigated the effect of the inclusion of antioxidant‐rich products or extracts in meat formulation on the oxidative phenomena during *in vitro* digestion. Trujillo‐Mayol *et al*.^35^ studied the effect of including a phenolic‐rich avocado peel extract in beef and soy‐based burgers on lipid oxidation during gastric digestion. When avocado peel extract was included at a concentration of 5 g kg^−1^, they found a decrease of 49% and 73% of TBARS after *in vitro* gastric digestion of beef and soy burgers, respectively. Similarly, the addition of chia seeds and goji puree in beef burgers decreased the amount of TBARS after intestinal digestion compared to control beef burgers.^36^ Furthermore, seasoning meat with herbs and spices, especially before cooking, led to a reduction in lipid hydroperoxides and lipid oxidation end‐products during *in vitro* gastro‐intestinal digestion.^32^, ^37^ The protective effect of spices on meat lipid oxidation was also confirmed *in vivo*. In fact, the addition of a spice mix to beef burgers decreased both plasmatic concentration and urinary excretion of malondialdehyde.^38^

### Identification of hazelnut skin extract phenolic compounds in raw and cooked meat and their bioaccessibility during *in vitro* digestion

The addition of 1% hazelnut skin extract to pork burger resulted in enrichment in phenolic compounds of meat as determined by HR‐MS analysis (Table 1). The concentration of phenolic compounds was 297.8 ± 8.7 μmol kg^−1^ meat in raw burgers. The percentage composition by classes was similar to that of the original hazelnut skin extract, with flavan‐3‐ols representing 81.8% of the total identified phenolic compounds (Table 1). The compounds present in the highest amount were epicatechin and catechin, followed by two isomers of procyanidin dimer B‐type. Meat cooking brought about a 10% increase in the concentration of total phenolic compounds, most likely as a result of water loss experienced during cooking. The phenolic profile of cooked burgers was similar to that of raw meat, with flavan‐3‐ols representing 82.3% of total phenolic compounds (Table 1). At the end of the gastric digestion, the amount of bioaccessible phenolic compounds was quite low, representing only about 6.8% (22.4 ± 2.8 μmol kg^−1^ meat) of phenolic compounds present in cooked burgers (Table 2). The majority of released phenolic compounds were hydroxybenzoic acids that represented 72.5% of the total phenolic compounds identified in the gastric digesta. A very low amount of flavan‐3‐ols was detected after gastric digestion with epicatechin being the only one identified. The low bioaccessibility of phenolic compounds in hamburgers during gastric digestion is likely the result of interactions with meat proteins or other meat constituents. Indeed, phenolic compounds may interact with meat proteins during gastro‐intestinal digestion by decreasing their bioaccessibility.^39^, ^40^

**Table 2:** Amount of phenolic compounds identified in pork burger samples formulated with HSGE after *in vitro* gastric and intestinal digestion.

Compound	Gastric digesta	Intestinal digesta	BI
	μmol kg^−1^ meat	μmol kg^−1^ meat	%
Hydroxybenzoic acid	8.0 ± 0.4 a	8.7 ± 1.0 a	102.4
Dihydroxybenzoic acid	0.2 ± 0.1 a	0.7 ± 0.1 b	43.8
Protocatechuic acid	5.5 ± 0.1 a	4.9 ± 0.1 b	104.3
Gallic acid	0.1 ± 0.0 a	4.7 ± 2.0 b	51.6
Galloyl‐shikimic acid isomer 1	ND	ND	0.0
Galloyl‐shikimic acid isomer 2	ND	ND	0.0
Gallic acid‐hexoside isomer 1	ND	0.3 ± 0.1	50.0
Gallic acid‐hexoside isomer 2	0.1 ± 0.0	ND	0.0
Gallic acid‐hexoside isomer 3	ND	ND	0.0
Gallic acid‐hexoside isomer 4	1.1 ± 0.3 a	1.2 ± 0.2 a	1200.0
Syringic acid‐4‐*O*‐hexoside isomer 1	ND	ND	ND
Syringic acid‐4‐*O*‐hexoside isomer 2	ND	ND	ND
Syringic acid‐4‐*O*‐hexoside isomer 3	1.1 ± 0.0 a	2.2 ± 0.4 b	75.9
*Total hydroxybenzoic acids*	*16.2 ± 1.1 a*	*22.6 ± 3.9 b*	*71.3*
Epicatechin	0.2 ± 0.2 a	15.9 ± 0.4 b	11.7
Catechin	ND	10.8 ± 0.3	13.8
Epigallocatechin	ND	ND	0.0
Gallocatechin	ND	ND	0.0
Epicatechin‐3‐*O*‐gallate	ND	2.8 ± 0.4	32.2
Catechin‐*O*‐hexoside	ND	ND	ND
Epigallocatechin‐3‐*O*‐gallate	ND	ND	0.0
Gallocatechin‐3‐*O*‐gallate	ND	ND	ND
Procyanidin dimer A type isomer 1	ND	ND	ND
Procyanidin dimer A type isomer 2	ND	ND	ND
Procyanidin dimer A type isomer 3	ND	ND	ND
Procyanidin dimer B type isomer 1	ND	2.5 ± 0.9	13.8
Procyanidin dimer B type isomer 2	ND	2.5 ± 0.5	15.2
Procyanidin dimer B type isomer 3	ND	1.0 ± 0.1	41.7
Catechin‐gallocatechin isomer 1	ND	ND	ND
Catechin‐gallocatechin isomer 2	ND	ND	ND
Procyanidin dimer B type gallate isomer 1	ND	ND	ND
Procyanidin dimer B type gallate isomer 2	ND	ND	ND
Procyanidin trimer B type	ND	ND	ND
Prodelphinidin trimer B type	ND	ND	ND
*Total flavan‐3‐ols*	*0.2 ± 0.2 a*	*35.4 ± 2.7 b*	*13.1*
Quercetin	ND	10.2 ± 1.3	108.5
Myricetin	ND	ND	0.0
Kaempferol‐3‐*O* rhamnoside	0.1 ± 0.0 a	0.5 ± 0.1 b	250.0
Quercetin‐3‐*O*‐rhamnoside	0.8 ± 0.2 a	5.3 ± 0.8 b	151.4
Myricetin‐3‐*O*‐rhamnoside	ND	0.7 ± 0.1	63.6
Quercetin‐3‐*O*‐rutinoside	ND	0.1 ± 0.0	100.0
Isorhamnetin‐3‐*O*‐rutinoside	ND	0.2 ± 0.0	200.0
*Total flavonols*	*0.9 ± 0.2 a*	*17.0 ± 2.4 b*	*105.6*
Phloretin 2′‐*O*‐glucoside	2.5 ± 0.6 a	8.7 ± 0.1 b	235.1
Ellagic acid	2.5 ± 0.7 a	10.2 ± 0.2 b	159.4
*Total others*	*5.0 ± 1.3 a*	*18.9 ± 0.3 b*	*185.3*
*Total phenolic content*	*22.4 ± 2.8 a*	*93.9 ± 9.3 b*	*28.7*

*Note*: ND, the compound was not detected in the sample. Different lowercase letters in the same row indicate that the values are significantly different (*P <* 0.05). Results are expressed in μmol kg^−1^ meat. Bioaccessibility index (BI) is the percentage ratio between the concentration after *in vitro* intestinal digestion and the concentration in the cooked pork burgers (Table 1).

Intestinal digestion increased the amount of bioaccessible phenolic compounds, reaching a value of 93.9 ± 9.3 μmol kg^−1^ meat, which represented 28.7% of total phenolic compounds found in cooked burgers (Table 2). The most representative class of phenolic compounds in intestinal digesta was flavan‐3‐ols, which accounted for 37.7% of total phenolic compounds. Despite this, flavan‐3‐ols presented a low bioaccessibility index of 13.1% (Table 2). Previous studies highlighted the low bioaccessibility index of oligomeric and monomeric flavan‐3‐ols.^41^, ^42^, ^43^ The high instability of oligomeric procyanidins during *in vitro* gastro‐intestinal digestion has been correlated to their hydrolysis into monomeric flavan‐3‐ols.^44^, ^45^ However, previous studies also found that the monomeric forms of flavan‐3‐ols underwent degradation during *in vitro* digestion, mainly via epimerization and auto‐oxidation reactions due to the residual dissolved oxygen and the elevated pH of the intestinal fluid.^41^, ^46^, ^47^

The bioaccessibility of hydroxybenzoic acids was higher than that of flavan‐3‐ols, with an intestinal recovery of 71.3% (Table 2). Hydroxybenzoic acid and protocatechuic acid were very stable under intestinal conditions, with a bioaccessibility index near 100%. The stability of protocatechuic acid under gastro‐intestinal conditions has already been observed.^48^ By contrast, gallic acid and gallic acid‐derivatives were less stable during *in vitro* digestion. As already reported, the low bioaccessibility of gallic acid‐hexosides may be a consequence of their deglycosylation occurring during *in vitro* digestion.^49^, ^50^, ^51^, ^52^ Furthermore, gallic acid may suffer from oxidative degradation in the intestinal milieu, producing unknown polymers with ellagic acid as an intermediate.^52^, ^53^ Accordingly, ellagic acid concentration increased by about 60% during *in vitro* digestion of hazelnut skin extract (Table 2).

The class of compounds with the highest bioaccessibility index was that of flavonols, with an intestinal recovery of about 100% (Table 2). Generally, flavonols, and especially the 3‐*O*‐glycosylated derivatives of quercetin, were considered to be unstable under alkaline intestinal conditions because of the oxidability of the catechol moiety in the B‐ring.^54^, ^55^, ^56^ The high bioaccessibility index of flavonols detected in this study may be the result of a food matrix effect. It can be speculated that the presence of meat may protect flavonols from degradation, resulting in a greater stability of these compounds. In particular, flavonols such as quercetin and its derivatives are known to interact via non‐covalent interactions with proteins present in meat, such as myofibrillar proteins.^39^ The protein–flavonols interaction may have protected flavonols from oxidation, resulting in high bioaccessibility. In this sense, Wang *et al*.^57^ demonstrated that the co‐digestion of some flavonoids, including flavonols, with bovine serum albumin and whey proteins resulted in a greater bioaccessibility compared to when the flavonoids were digested in the absence of proteins. Furthermore, Tarko *et al*.^58^ found that flavonols were much more stable during the *in vitro* digestion of whole fruit compared to the *in vitro* digested corresponding food extract, suggesting that fruit matrix may protect these compounds from degradation.

## CONCLUSIONS

HSGE is an important source of phenolic compounds especially monomeric and oligomeric flavan‐3‐ols, characterized by high antioxidant activity.

The inclusion of HSGE in pork burgers exhibited a preventive effect towards lipid oxidation, mitigating and hindering the formation of both lipid hydroperoxides and advanced lipid oxidation end‐products, either during cooking or *in vitro* gastro‐intestinal digestion. The reported inhibitory effects against lipid oxidation are ascribable to the presence of hazelnut skin‐derived phenolic compounds in raw and cooked meat. During *in vitro* gastro‐intestinal digestion, phenolic compounds were released from meat, further protecting meat lipids from the oxidative degradation. Most phenolic compounds were stable during *in vitro* gastro‐intestinal digestion, suggesting a positive food matrix effect due to the presence of meat.

Incorporating by‐products such as hazelnut skin into pork burgers helps reduce food waste and disposal costs at the same time as improving meat quality and human health by minimizing lipid oxidation and harmful compound production during cooking and digestion. Moreover, hazelnut skin‐formulated pork burgers may represent an unconventional source of bioaccessible and potentially bioavailable phenolic compounds with demonstrated protective effects on human health.

## FUNDING

This research was funded by PRIN 2020 National Project (Grant N. 2020244EWW).

## CONFLICTS OF INTEREST

The authors declare that they have no conflicts of interest.

## Supporting information


**Table S1.** Mass spectrometry data of phenolic compounds identified in hazelnut skin green extract and characteristic of the standard compounds used for quantitfication.

## Data Availability

The data that support the findings of this study are available from the corresponding author upon reasonable request.
